# A Four-Way Comparison of Cardiac Function with Normobaric Normoxia, Normobaric Hypoxia, Hypobaric Hypoxia and Genuine High Altitude

**DOI:** 10.1371/journal.pone.0152868

**Published:** 2016-04-21

**Authors:** Christopher John Boos, John Paul O’Hara, Adrian Mellor, Peter David Hodkinson, Costas Tsakirides, Nicola Reeve, Liam Gallagher, Nicholas Donald Charles Green, David Richard Woods

**Affiliations:** 1 Department of Cardiology, Poole Hospital NHS Foundation trust, Poole, United Kingdom; 2 Dept of Postgraduate Medical Education, Bournemouth University, Bournemouth, United Kingdom; 3 Research Institute, for Sport, Physical Activity and Leisure, Leeds Beckett University, Leeds, United Kingdom; 4 James Cook University Hospital, Middlesbrough, TS4 3BW, United Kingdom; 5 Defence Medical Services, Lichfield, United Kingdom; 6 RAF Centre of Aviation Medicine, RAF Henlow, Beds, SG16 6DN, United Kingdom; 7 Division of Anaesthesia, University of Cambridge, Box 93, Addenbrooke’s Hospital, Hills Road, Cambridge CB2 2QQ, United Kingdom; 8 Northumbria and Newcastle NHS Trusts, Wansbeck General and Royal Victoria Infirmary, Newcastle, United Kingdom; 9 University of Newcastle, Newcastle upon Tyne, United Kingdom; University of Southampton, UNITED KINGDOM

## Abstract

**Background:**

There has been considerable debate as to whether different modalities of simulated hypoxia induce similar cardiac responses.

**Materials and Methods:**

This was a prospective observational study of 14 healthy subjects aged 22–35 years. Echocardiography was performed at rest and at 15 and 120 minutes following two hours exercise under normobaric normoxia (NN) and under similar PiO_2_ following genuine high altitude (GHA) at 3,375m, normobaric hypoxia (NH) and hypobaric hypoxia (HH) to simulate the equivalent hypoxic stimulus to GHA.

**Results:**

All 14 subjects completed the experiment at GHA, 11 at NN, 12 under NH, and 6 under HH. The four groups were similar in age, sex and baseline demographics. At baseline rest right ventricular (RV) systolic pressure (RVSP, p = 0.0002), pulmonary vascular resistance (p = 0.0002) and acute mountain sickness (AMS) scores were higher and the SpO_2_ lower (p<0.0001) among all three hypoxic groups (GHA, NH and HH) compared with NN. At both 15 minutes and 120 minutes post exercise, AMS scores, Cardiac output, septal S’, lateral S’, tricuspid S’ and A’ velocities and RVSP were higher and SpO_2_ lower with all forms of hypoxia compared with NN. On post-test analysis, among the three hypoxia groups, SpO_2_ was lower at baseline and 15 minutes post exercise with GHA (89.3±3.4% and 89.3±2.2%) and HH (89.0±3.1 and (89.8±5.0) compared with NH (92.9±1.7 and 93.6±2.5%). The RV Myocardial Performance (Tei) Index and RVSP were significantly higher with HH than NH at 15 and 120 minutes post exercise respectively and tricuspid A’ was higher with GHA compared with NH at 15 minutes post exercise.

**Conclusions:**

GHA, NH and HH produce similar cardiac adaptations over short duration rest despite lower SpO_2_ levels with GHA and HH compared with NH. Notable differences emerge following exercise in SpO_2,_ RVSP and RV cardiac function.

## Introduction

Hypoxic exposure has a number of important clinical applications. These include pre-acclimatization training for athletes, the investigation of high altitude (HA) illnesses such as acute mountain sickness (AMS) and clinical diseases complicated by tissue hypoxia [1,2]. In order to improve the understanding of the clinical effects of genuine HA, hypoxia has been experimentally reproduced typically using normobaric hypoxia (NH) or hypobaric hypoxia (HH). NH lowers the partial pressure of inspired oxygen (PiO_2_) by reducing the fraction of inspired oxygen (FiO_2_) through addition of exogenous nitrogen (N_2_) without altering the barometric pressure whereas HH lowers the Pio_2_ by reduction of barometric pressure [2].

There has been considerable and ongoing debate in the medical literature as to whether these differing methods of hypoxic challenge are meaningfully different or clinically important and most importantly whether they are effective surrogate for real life HA [2–8]. A large number of the comparative studies have been in animal models and the human studies have been predominantly two-way comparisons of NH with HH exposure and have not included either a genuine ‘real-world’ terrestrial HA (GHA) or a normobaric normoxia control (NN) group, akin to normal sea level, limiting the clinical impact of their findings [2]. It has become increasingly appreciated that the physiological responses to GHA at a given altitude are influenced by variations in the ambient barometric pressure due to the differences in latitude, time of year and prevailing weather conditions which may be an important factor in comparative studies [3,9]. Hence, it is crucial that the ambient pressure for a given field altitude is documented in to allow more reliable comparison both between GHA experiments and with HH chamber studies [3,9]. Most of the previously published studies to compare differing hypoxia modalities have been undermined by their relatively short periods of hypoxic exposure (<30 minutes), the use of a separate but matched populations for the differing hypoxic challenge groups and the use of only brief wash out periods between each exposure increasing the risk of acclimatization bias [2]. Furthermore, the important stimulus of exercise which is a crucial factor in the majority of HA ventures in real life has been frequently overlooked.

Acute hypoxia leads to a number of recognized cardiopulmonary responses, which notably includes pulmonary vasoconstriction and an associated increase in pulmonary vascular resistance [1, 10–13]. Whether HA and the associated hypoxia leads to deleterious effects on cardiac function remains still remains a controversial issue [2,8]. Published studies have consistently shown that acute hypoxia leads to an increase in resting cardiac output and preservation of long axis systolic and radial systolic function [2, 9–12]. However, more concerning, there is a blunted stroke volume response and variable effects on left ventricular diastolic filling and right ventricular systolic function have been observed [9,11,13]. Right ventricular (RV) diastolic function has been barely explored [2,10]. Evidence to suggest the potential deleterious effects of HA on cardiac performance include the observed increase in brain natriuretic peptide (BNP) levels at HA compared with sea level and their link to AMS and its severity [10,14,15]. It has also been shown that sustained hypoxia can lead to a decline in cardiac energetics, which is linked to adverse changes in left ventricular diastolic function despite preservation of systolic function [16].

There have been only two studies to date that have tried to compare potential changes in cardiac function during exercise following differing modes of hypoxic challenge and in both echocardiographic assessments of biventricular performance and/or right ventricular systolic pressure were not assessed [17,18]. A four-way comparison of NN, genuine HA (GHA), NH and HH on cardiac function has never been performed. Furthermore, none of the cross comparison studies to date utilized recent advances in echocardiography allowing much detailed assessment of biventricular systolic and diastolic function. Consequently, in this study we sought to investigate, for the first time, the effects of acute and sustained hypoxia on cardiac function at rest and following exercise under NN, NH and HH and GHA.

## Materials and Methods

### Study population

This was a prospective observational study of 14 healthy British military servicemen aged 22–35 years. In addition to completing a detailed health questionnaire all subjects were required to have a normal baseline ECG and echocardiogram to confirm suitability for inclusion. Baseline health status was undertaken via a history, clinical examination, bloods, electrocardiogram and transthoracic echocardiogram.

### Study protocol

All participants completed a standard maximal incremental cycle test to volitional exhaustion at sea level (absolute altitude ~113m) under normobaric normoxia (NN) to determine maximal oxygen uptake and maximal workload (Wmax [watts]) [19]. This was followed by a maximal incremental test to volitional exhaustion >24 hours later under NH (an FiO_2_ equivalent to 3375m/11078ft (PiO_2_ ~95 mmHg) in order to establish and ensure equivalent workloads for the hypoxic experimental trials [19].

Participants were then required to complete physiological assessments prior to and during exercise and rest under four different conditions. They were then assessed at GHA at (3375m/11078ft, 'real' altitude, barometric pressure 506.4 ± 1.7 mmHg), followed in order with assessments at NN, NH (TISS, Alton, UK and Sporting Edge, Sherfield on Loddon, UK) and HH (Centre for Aviation Medicine, RAF Henlow, Henlow, UK) ensuring a minimum washout period of >7 days between each experimental condition. This sequence ensured the PiO_2_ experienced breathing ambient air during GHA (PiO_2_ = 96.3 ± 0.4 mmHg) could be replicated for each individual during subsequent NH and HH exposures.

For NH the Fi0_2_ (13.9 ± 0.2%) was manipulated to equate to each individuals Pi0_2_ established at terrestrial GHA using the following equation, which considers fluctuations in sea level barometric pressure [3,8]:

Fi02=PiO2(mmHg)/localPB(mmHg)−PH20(47mmHg)

For the measurements in the HH chamber participants underwent a decompression period (0.33 mmHg s^-1^ equivalent to 5 m.s^-1^ (ascent rate) ~10 minutes) to the target altitude, recreating each participants PiO_2_, and on completion of the exposure a recompression period (0.33 mmHg s^-1^) to the ambient pressure. The chamber was continuously flushed with medical quality gas to maintain the inspired fractions of O_2_ and CO_2_ at 20.9% and 0.03%, respectively, with nitrogen balance. During the HH exposure participants were continuously monitored by a chief medical officer, who was present in the chamber breathing through an O_2_ diluter demand mask. They were in constant contact with the chamber operators and additional medical staff.

The GHA challenge involved rapid ascent by cable car to 3375m after the subjects were driven in a minibus from sea level to 1400m. The NN and NH experiments were undertaken within a NH chamber at Leeds Beckett University and the HH chamber at the Royal Air Force Centre of Aviation Medicine, Henlow.

Each experiment was performed following a 12-hour overnight fast. All subjects underwent 30 minutes of altitude acclimatization followed by a complete 120 minutes of cycling exercise (a progressive intensity warm-up for 15 minutes, followed by 105 minutes at 55% Wmax based on the NH maximal exercise test). All exercising testing occurred on a bicycle affixed to a bicycle trainer (Compu Trainer Pro Lab, Racer Mate, USA). The cycle ergometer was calibrated following the manufacturer’s instructions. The load generator ensured the relative workloads between conditions for each participant were accurately maintained, taking into consideration an individual’s natural torque fluctuations with each pedal stroke. The manufacturer’s reports an accuracy of 2.5% and repeatability of 1%. Each experimental trial involved the ingestion of a carbohydrate solution (glucose-fructose) so that the dietary intake of each participant was standardised across all four studies. Physiological measurement of AMS scores, haemodynamics and cardiac function were assessed at rest, at 15 minutes into the rested acclimatization process in each study condition and then again rested 15 minutes and 120 minutes post two hours of cycling exercise in the hypoxic environment. A consistent temperature range of 18–23°C was maintained for all four study conditions.

### Ethics

The study was approved by the Ministry of Defence Research and Medical Ethics Committee and was conducted according to the standards of the declaration of Helsinki and all subjects underwent written informed consent.

### Physiological measurements

Resting recordings of oxygen saturations (SpO2) were performed using a Nellcor N-20P pulse oximeter (Nellcor Puritan Bennett, Coventry, UK) following a 15 second continuous recording using the index finger of the right hand with the most consistent reading being used. Blood pressure were measured using an automated blood pressure cuff with the subject sat upright for >10 minutes at rest M6 (Omron Healthcare, Milton Keynes, UK) and heart rate was measured form a single lead ECG at the time of the echocardiogram. The ambient temperature was recorded for each experimental condition (NN.NH, HH and GHA) using a PCE-THB 40 Barometer (PCE Instruments UK Ltd).

### Acute mountain sickness (AMS) scores

HA related symptoms were assessed using the Lake Louis Scoring System (LLS) [20]. The LLS score allocates a score of 0–3 (symptom not present to severe) for symptoms of AMS (headache, gastrointestinal symptoms, fatigue/weakness, dizzy/light-headedness, difficulty sleeping). A total score of ≥3 in the presence of a headache is consistent with AMS and ≥6 with severe AMS [15,20].

### Echocardiographic assessment

All echocardiographic assessments were undertaken using a portable Vivid I echocardiogram machine (GE Healthcare™, Amersham, Bucks, UK) with a 1.5–3.6 MHz S4 transducer. Pulsed-wave and two dimensional colour images were acquired in the parasternal short axis and apical four-chamber view during a short end-expiration pause with the subject lying in the left lateral position. RVSP was estimated from the maximum velocity of the trans-tricuspid gradient using continuous wave Doppler imaging [16,21]. The pulsed-wave sample volume of the conventional Doppler was placed at the tips of the mitral and tricuspid valve leaflets in order to measure the peak early transvalvular flow velocity (E), and the peak flow velocity (A) of late diastolic filling and the E/A ratios [22]. Pulsed-wave TDI volume samples were recorded at the septal and lateral mitral annulus and over the RV free wall to assess early and late diastolic filling due to left ventricular relaxation (E’) and atrial contraction (A’) and long axis systolic function (S’) (10–12,22). The pulmonary artery vascular resistance (PVR) was calculated using the following equation *PVR = 80 x TRV/VTI RVOT* where TRV was the maximal tricuspid regurgitation velocity and velocity time integral of the RV outflow tract velocity measured using pulsed wave doppler at the level of the pulmonary valve in the parasternal short axis view as previously described [10].

Pulsed-wave TDI was used to quantify the respective left and right ventricular isovolumic contraction (ICT) and isovolumic relaxation times (IRT) and the isovolumic contractile velocities (ICV) [3]. Right and left ventricular myocardial performance (Tei) indices (IRT+ICT/ejection time) were performed using TDI [10, 23]. Tricuspid annular plane systolic excursion (TAPSE) was recorded using M-Mode as previously described [24]. The isovolumic contractile velocity was measured at the tricuspid and mitral annulus using PWTDI [21,22]. Stroke volume and cardiac output were calculated using the aortic systolic flow velocity integral, using pulsed-wave profile of aortic blood flow from the apical five chamber view and the cross sectional area of the Left ventricular outflow tract [11,12]

### Statistical Methods and power calculations

Data were analysed using SPSS^®^ statistics version 22. The Kolmogorov-Smirnov test and inspection of the data was undertaken to assess normality of all continuous data. All data are presented as mean ± standard deviations. Between group comparisons of categorical data for three or more groups were compared using the Fisher’s Exact Test. Continuous data across the four experimental altitude groups (NN, HA, NH and HH) were assessed using Ordinary ANOVA with Bonferroni post-test for parametric data and with Kruskal-Wallis and Dunn Post-test for non-parametric data when the P value was <0.05. Time dependent changes (rest, 15 and 120 minutes post exercise) of continuous data within each group were assessed using Repeated measures ANOVA with Bonferroni post-test for parametric data and using Friedman Test with Dunn Post-test for non- parametric data. Correlation was assessed using Spearman Rank correlation with the 95% confidence interval of R. Further exploratory analyses of the three hypoxia groups only were undertaken using a Two-Way split level 3x3 Repeated Measures ANOVA. The within-subjects main effect of time (before and 15 and 120 min after exercise) and the between-subject main effects mode of hypoxia (GHA, NH and HH) with Bonferroni post-tests and their interactions (and effect size, Eta [*n*^*2*^]) were assessed. A two tailed *P* value <0.05 was considered statistically significant for all comparisons.

Sample size calculations were based on previous studies. In 11 out of the 13 prior comparative experimental hypoxia studies the sample size has been between 7–12 subjects [2]. In another very recent comparative study of six subjects Beidleman et al observed that cycling time trial performance was impaired to a greater degree in HH versus NH at the same ambient PO_2_ equivalent to 4,300 m despite similar cardiorespiratory responses [17]. Hence, based on this previously published work it was calculated that a sample size of ≥12 subjects would be sufficient to detect a significant difference in cardiac performance and allow for a minimum group sample size of 6 subjects in the event of any drop outs given the intense and prolonged nature of these four group comparisons.

## Results

Fourteen subjects completed the genuine HA phase, 11 the sea level study, 12 with NH and 6 under HH. All subjects completed the exercise task in each group. Non-completion was mainly due to inter-current illness and in the case of HH failure to clear their ears or voluntary withdrawal. There were no significant differences in any of the baseline demographics across the four groups with similar ages, sex, height, and body weight and body mass indices (Table 1; S1 and S2 Tables).

**Table 1:** Baseline demographics.

Variable	NN	GHA	NH	HH	P value
Number	11	14	12	6	
Age, years	26.4 ± 4.0	25.9 ± 3.8	26.1 ± 4.1	26.3 ± 3.8	0.96
Range	22–35	21–35	21–35	22–33	
Males (%)	7 (64%)	8 (57%)	8 (67%)	4 (67%)	0.99
Height, m	175.4 ± 9.7	174.4 ± 9.6	175.5 ± 10.0	178.3 ± 9.7	0.88
Weight, kg	72.5 ± 8.7	71.5 ± 9.9	72.9 ± 10.1	72.8 ± 11.4	0.99
BMI, kg/m^2^	23.5 ± 1.9	23.4 ± 1.90	23.6 ± 2.0	22.8 ± 1.8	0.90
Current smokers n,%	6 (54.5%)	7 (50%)	5 (35.7%)	2 (33.3%)	0.89
Blood haemoglobin g/dL	14.5 ± 1.6	14.5 ± 1.7	14.7 ± 1.5	15.2 ± 1.4	0.80
Temperature, °C	20.8±1.7	19.1±1.4	19.3±0.5	22.1±2.0	0.008a,b

NN, normobaric hypoxia; GHA, genuine high altitude; NH, normobaric hypoxia; HH, hypobaric hypoxia

Post-test differences: a, NH vs HH; b, GHA vs HH

### Physiological and haemodynamic indices

The mean peak oxygen consumption (VO_2_) at baseline with NN was 46.3±5.7mls/kg/minute. There was no difference in the mean peak VO_2_ among the subjects who were subsequently included in the GHA, NH and HH groups respectively (46.7±5.7, 46.2±5.2 and 47.2±4.7 mls/kg/minute; P = 0.98). As expected peak VO_2_ was significantly lower under NH versus NN (38.9± vs 46.3±5.7 mls/kg/minute; P = 0.007).

The ambient temperature was marginally but significantly higher with HH versus NH and HH (Table 1). There was a significant reduction in SpO_2_ across all three hypoxia environments (GHA, NH and HH) compared with NN (P<0.001), which was sustained at all three time points (rest, 15 minutes and 120 minutes post exercise) (Table 2). On Post-test analysis SpO_2_ was higher at baseline and at 15 minutes post exercise with GHA (89.3±3.4% and 89.3±2.2%) and HH (89.0±3.1 and (89.8±5.0) compared with NH (92.9±1.7 and 93.6±2.5%) (Table 2). AMS scores were higher at all time points in the three hypoxic conditions versus NN with no between group differences among the hypoxia groups. Absolute resting heart rates were higher across all three hypoxic group time points compared with NN (Table 2). At 120 minutes post exercise, resting heart rates were significantly greater with GHA than NH.

**Table 2:** Changes in acute mountain sickness (AMS) and haemodynamic variables.

Variable	NN	GHA	NH	HH	P Value
Lake Louise Scores					
-Rest	0	1 ± 1.0	1.4 ± 2.3	0.7 ± 1.2	0.02^ab^
-15 minutes post exercise	0	2.7 ± 3.0	2.6 ± 2.5	2.7 ± 3.4	0.004^ab^
-2 hours post exercise	0	2.3 ± 2.2	2.1 ± 2.1	2.7 ± 3.0	0.004^ab^
Heart Rate, minute^-1^					
-Rest	60.1 ± 9.9	72.1 ± 9.0	65.7 ± 10.6	65.7 ± 7.5	0.02^a^
-15 minutes post exercise	79.9 ± 11.1*	89.7 ± 8.6*	86.6 ± 9.9*	88.7 ± 8.7*	0.09
-2 hours post exercise	67.5 ± 9.4**	89.4 ± 10.0**	76.6 ± 15.3**	80.2 ± 11.6**	0.0005^a,d^
Oxygen Saturations, %					
-Rest	98.6 ± 1.4	89.3 ± 3.4	92.9 ± 1.7	89.0 ± 3.1	<0.001^abcdf^
-15 minutes post exercise	98.4 ± 1.4	89.3 ± 2.2	93.6 ± 2.5	89.8 ± 5.0	<0.01^abcdf^
-2 hours post exercise	98.9 ± 1.5	91.6 ± 3.1**	92.8 ±4.3	91.8 ± 5.2	<0.01^abc^

NN, normobaric normoxia; GHA, genuine high altitude; NH, normobaric hypoxia; HH, hypobaric hypoxia; LLS, Lake Louis Scores; AMS-C, Acute Mountain Sickness Scores; Post-test differences: a, NN vs GHA; b, NN vs NH; c, NN vs HH; d, GHA vs NH; e, GHA vs HH; f, NH vs HH

* rest vs 15 minutes post exercise

** rest vs 2 hours post exercise

### Echo parameters of left ventricular function

**Fig 1:**
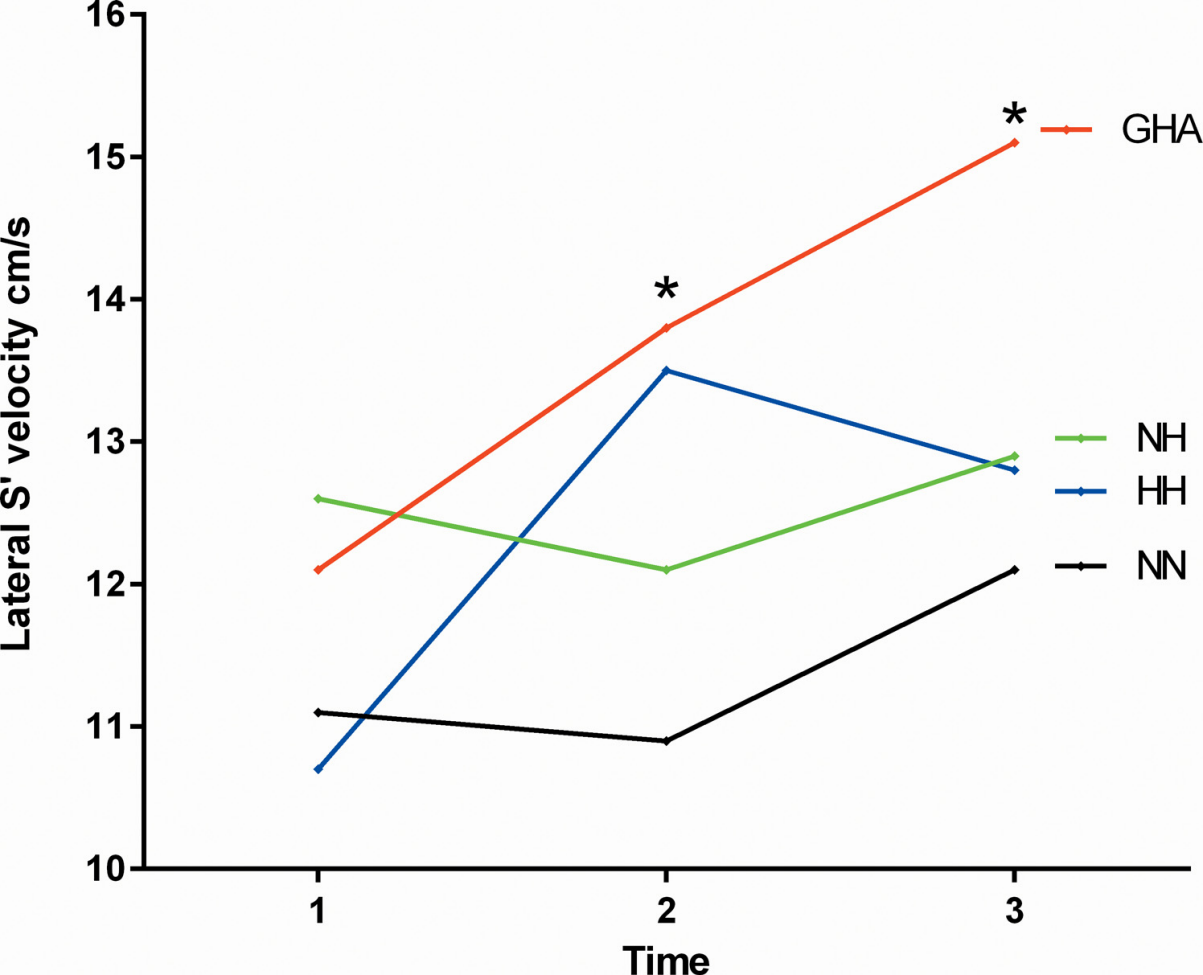
Changes in the left ventricular lateral S’ velocities (marginal means) with differing experimental conditions and duration of hypoxia (time 1 = baseline rest, time 2 = 15 minutes post exercise and time 3 = 120 minutes post exercise). * demonstrates between group differences on post test.

There were no differences in any of the echo parameters of left ventricular systolic or diastolic function at rest across the four groups. However, at 15 minutes post exercise Cardiac output (p = 0.01), septal S’ (p = 0.02) and lateral S’ (p = 0.03) and septal A’ (p = 0.003) velocities were higher with all forms of hypoxia compared with NN with no differences between the hypoxic groups (Table 3). At 120 minutes post exercise cardiac output (p = 0.006), septal S’ (p = 0.04), mitral A (p = 0.009) and lateral S’ (p = 0.02) velocities were higher with acute hypoxia versus NN, with no intergroup differences among the three hypoxia groups (Table 3; Fig 1). Exercise led to an increase in the post exercise left ventricular myocardial performance (Tei) index across all four groups, which was significant at 15 minutes post exercise in the NN, NH and HH groups and only in the HH at 120 minutes post exercise compared with baseline rest (Table 3). Compared with baseline, stroke volume fell at 15 minutes post exercise under all four experimental conditions before increasing again in all except the HH group where the fall in stroke volume was sustained.

**Table 3:** Changes in Echo derived markers of Left ventricular function.

Variable	NN	GHA	NH	HH	P Value
Stroke volume, ml					
-Rest	71.5 ± 11.9	74.1 ± 11.7	73.6 ± 13.6	74.7 ± 8.4	0.85
-15 minutes post exercise	60.6 ± 8.2 *	62.7 ± 8.5*	66.5 ± 11.5 *	69.8 ± 8.5	0.16
-2 hours post exercise	66.1 ± 10.2	66.6 ± 9.3**	70.9 ± 12.6	69.7 ± 9.5	0.58
Cardiac output, L/minute					
-Rest	4.3 ± 0.8	5.3 ± 1.1	4.9 ± 1.3	4.9 ± 1.0	0.24
-15 minutes post exercise	4.8 ± 0.8	5.6 ± 1.0	5.9 ± 1.1*	6.2 ± 0.9*	0.01^b,c^
-2 hours post exercise	4.4 ± 0.5	5.9 ± 1.1	5.4 ± 1.2	5.6 ± 0.9	0.006^a^
Mitral E velocity, cm/s					
-Rest	88.6 ± 9.6	90.1 ± 12.5	92.5 ± 18.2	95.2 ± 11.7	0.78
-15 minutes post exercise	72.0 ± 16.2*	81.8 ± 13.2	77.4 ± 15.2*	84.5 ± 18.5	0.19
-2 hours post exercise	76.5 ± 15.0	85.4 ± 14.0	79.8 ± 13.3**	76.7 ± 17.6 **	0.48
Mitral A velocity, cm/s					
-Rest	48.5 ± 14.4	51.8 ± 9.2	55.2 ± 11.0	58.2 ± 11.4	0.39
-15 minutes post exercise	56.7 ± 9.5	63.3 ± 17.3	68.5 ± 12.1*	64.3 ± 11.0	0.23
-2 hours post exercise	46.6 ± 12.4	59.9 ± 14.8	67.8 ± 16.7 **	62.0 ± 10.1	0.009^b^
Mitral E/A ratio					
-Rest	1.71 ± 0.27	1.79 ± 0.42	1.71 ± 0.37	1.67 ± 0.26	0.87
-15 minutes post exercise	1.08 ± 0.25*	1.39 ± 0.45*	1.15 ± 0.26*	1.35 ± 0.36	0.13
-2 hours post exercise	1.25 ± 0.42**	1.53 ± 0.55	1.26 ± 0.42 **	1.27±0.24*	0.32
Septal S’ velocity, cm/s					
-Rest	8.9 ± 1.4	10.2 ± 1.4	9.8 ± 1.6	9.0 ± 0.9	0.33
-15 minutes post exercise	8.3 ± 1.2	10.1± 1.7	9.4 ± 1.2	9.8 ± 0.8	0.02^a^
-2 hours post exercise	8.9 ± 1.4	10.9 ± 1.7	9.8 ± 1.2	9.7 ± 1.2	0.04^a^
Septal E’ velocity, cm/s					
-Rest	13.0 ± 1.6	12.9 ± 1.9	13.3 ± 3.3	13.2 ± 1.5	0.97
-15 minutes post exercise	10.8 ± 1.5*	12.9 ± 1.9	12.7 ± 3.0	12.7 ± 1.5	0.11
-2 hours post exercise	11.6 ± 1.6**	12.7 ± 2.5	12.0 ± 2.0	11.0 ± 1.8**	0.36
Septal A’ velocity, cm/s					
-Rest	7.9 ± 2.3	8.6 ± 2.2	9.6 ± 2.2	8.3 ± 2.0	0.09
-15 minutes post exercise	8.2 ± 1.2	11.2 ± 3.4*	11.1 ± 2.2*	10.8 ± 2.3*	0.003^abc^
-2 hours post exercise	8.5 ± 1.8	10.7 ± 3.1**	10.9 ± 2.6	10.0 ± 1.1	0.11
Septal ICV, cm/s					
-Rest	6.5 ± 1.8	7.1 ± 1.6	7.0 ± 2.1	6.8 ± 1.3	0.80
-15 minutes post exercise	7.1 ± 2.1	8.4 ± 2.9	7.2 ± 2.2	6.8 ± 2.4	0.55
-2 hours post exercise	7.2 ± 1.9	8.8 ± 3.2	7.3 ± 2.1	7.7 ± 2.2	0.46
Lateral S’ velocity, cm/s					
-Rest	11.1 ± 2.2	12.1 ± 2.5	12.6 ± 2.4	10.7 ± 1.7	0.06
-15 minutes post exercise	10.9 ± 1.4	13.8 ± 2.8	12.1 ± 2.4	13.5 ± 2.2*	0.03^a^
-2 hours post exercise	12.1 ± 2.3	15.1 ± 2.9**	12.9 ± 2.3	12.8 ± 1.6**	0.02^a^
Lateral E’ velocity cm/s					
-Rest	18.3 ± 3.1	18.6 ±2.1	20.8 ± 2.8	17.8 ± 2.8	0.90
-15 minutes post exercise	16.5 ± 2.8	17.9 ± 3.2	17.2 ± 2.5*	15.5 ± 1.6	0.52
-2 hours post exercise	17.4 ± 2.7	18.0 ± 3.8	18.1 ± 3.2**	16.0 ± 2.3	0.57
Lateral A’ velocity cm/s					
-Rest	7.9 ± 2.5	9.4 ± 2.8	9.0 ± 2.6	7.7 ± 2.2	0.30
-15 minutes post exercise	8.4 ± 2.3	10.6 ± 2.4	10.1 ± 2.5	10.5 ± 2.2	0.13
-2 hours post exercise	8.9 ± 2.2	10.7 ± 3.5	9.9 ± 3.2	9.8 ± 2.9	0.54
Lateral ICV, cm/s					
-Rest	8.1 ± 2.0	9.1 ± 2.8	7.6 ± 2.0	7.7 ± 1.6	0.34
-15 minutes post exercise	8.6 ± 1.8	9.6 ± 3.0	9.2 ±2.5	10.7 ± 3.1*	0.52
-2 hours post exercise	9.2 ± 1.8	8.9 ± 3.6	9.5 ± 2.5**	8.8 ± 1.8	0.94
Left ventricular Tei Index					
-Rest	0.28 ± 0.04	0.30 ± 0.03	0.29 ± 0.04	0.29 ± 0.03	0.59
-15 minutes post exercise	0.33 ± 0.04*	0.32 ± 0.04	0.33 ± 0.03*	0.37 ± 0.06*	0.15
-2 hours post exercise	0.32 ± 0.04	0.33 ± 0.03	0.33 ± 0.03	0.36 ± 0.02**	0.13

NN, normobaric normoxia; GHA, genuine high altitude; NH, normobaric hypoxia; HH, hypobaric hypoxia; ICV, isovolumic contractile velocity; Between group post-test differences: a, NN vs GHA; b, NN vs NH; c, NN vs HH; Within groups repeated measures post-test difference

* rest vs 15 minutes post exercise

** rest vs 2 hours post exercise

### Echo parameters of right ventricular function

**Fig 2:**
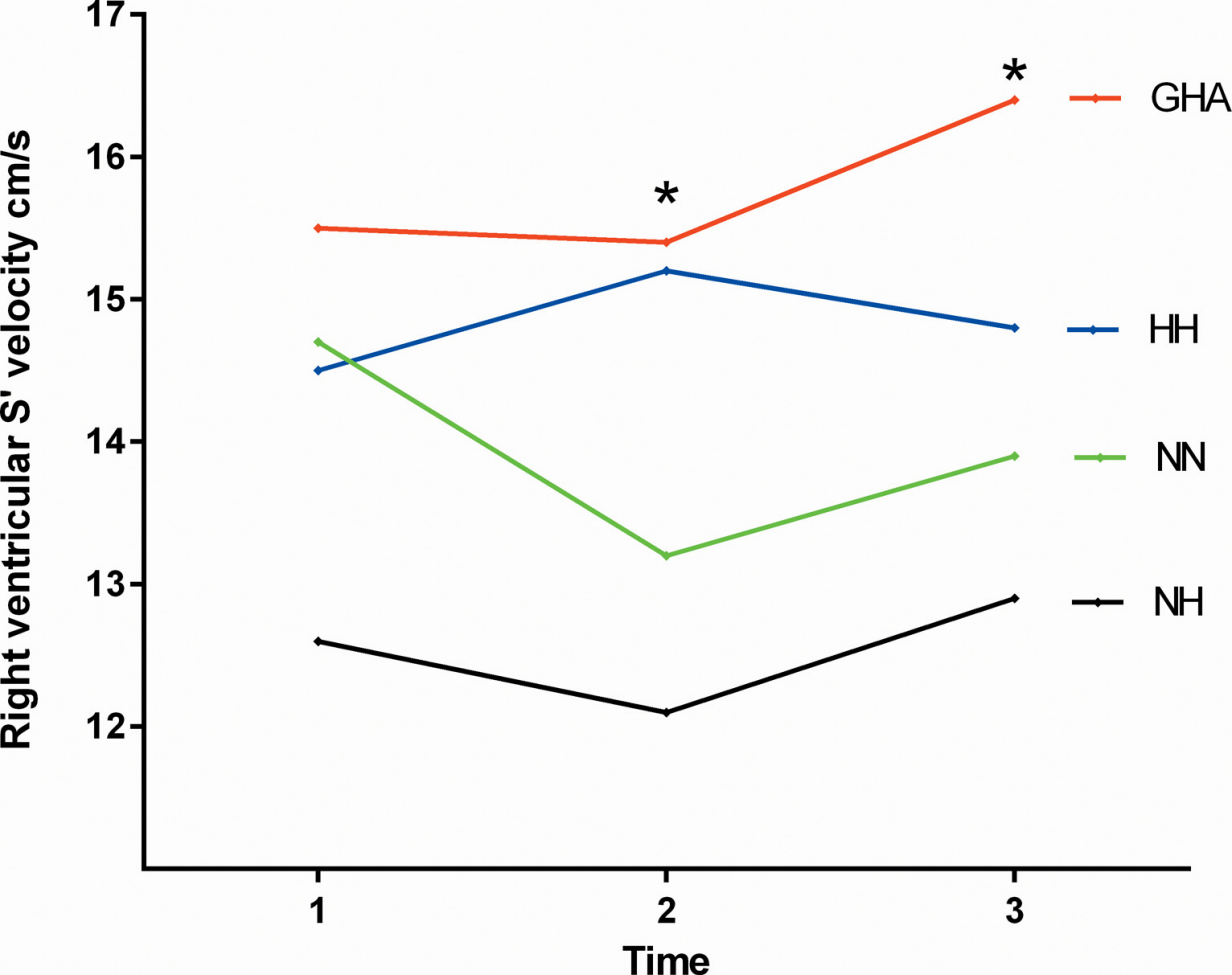
Changes in the right ventricular S’ velocities (marginal means) with differing experimental conditions and duration of hypoxia (time 1 = baseline rest, time 2 = 15 minutes post exercise and time 3 = 120 minutes post exercise). * demonstrates between group differences on post test.

**Fig 3:**
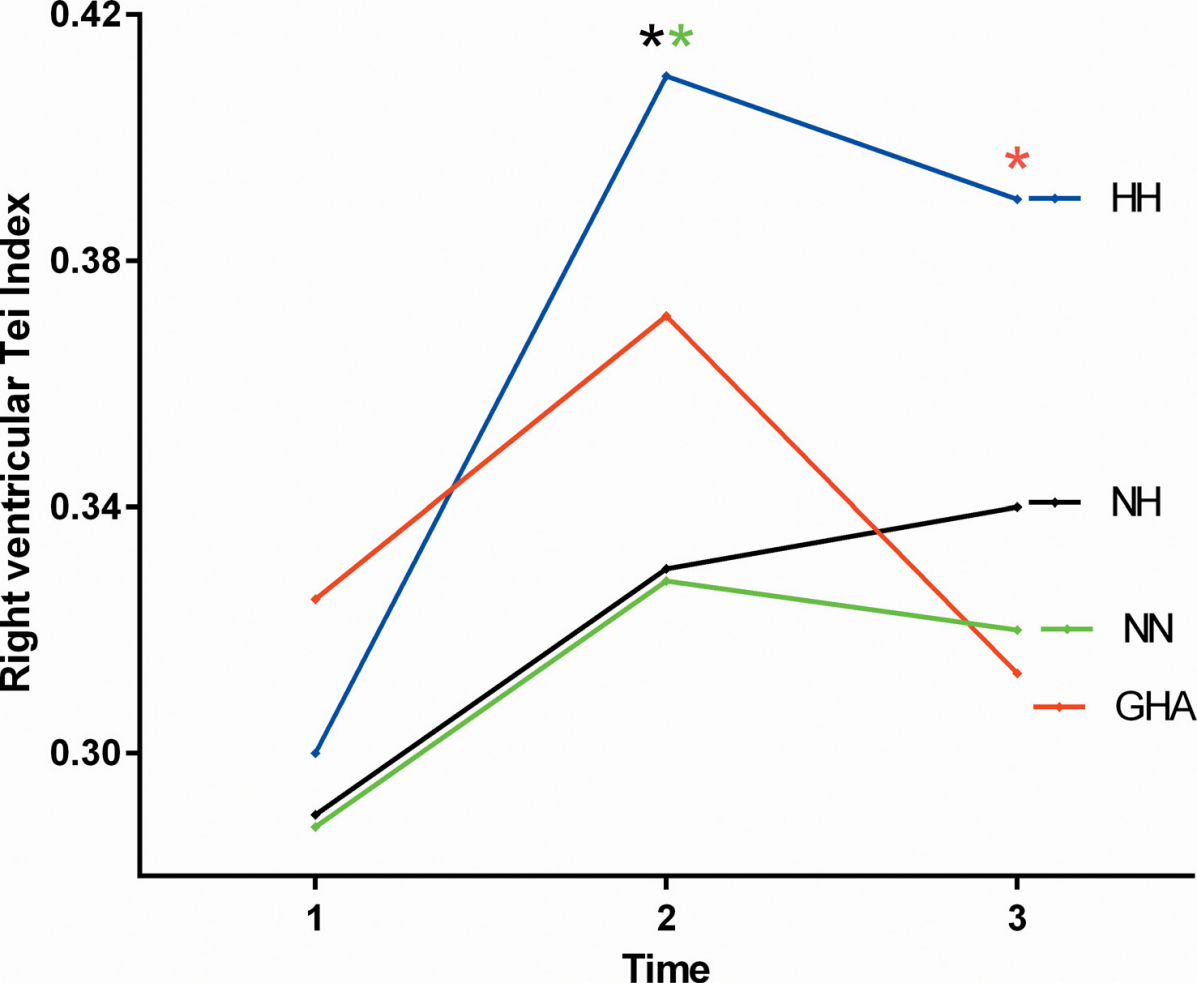
Changes in the right ventricular Tei Index (marginal means) with differing experimental conditions and duration of hypoxia (time 1 = baseline rest, time 2 = 15 minutes post exercise and time 3 = 120 minutes post exercise) * demonstrates between group differences on post test.

Resting RVSP (p = 0.0002), PVR (p = 0.0002) and tricuspid A velocities (p = 0.01) were higher with all forms of hypoxia compared with NN with no between group differences among the three hypoxia groups (Table 4). Compared with NN at 15 minutes post exercise RVSP (p = 0.04), Tricuspid S’ (p = 0.009) and A’ (p = 0.0001) velocities and the right ventricular Tei Index (0.001) were greater with hypoxia. The tricuspid A’ velocity was significantly higher with GHA and the Tei index and RVSP with HH on post-test analysis. At 120 minutes post exercise the RVSP (p = 0.0006), Tricuspid A (p = 0.006), tricuspid S’ (p = 0.03) and A’ (p = 0.0007) velocities and the RV Tei index (p = 0.004) were higher with hypoxia than NN (Figs 2 and 3). The rise in tricuspid A’ velocity, RV Tei index and RVSP were greatest among the GHA and HH groups respectively on post-test analysis (Table 4). There was a trend for higher PVR with HH than with either NH or GHA at all three sampling time points (p<0.05 for trend). There was a significant correlation between pulmonary vascular resistance and the RV Tei index (r = 0.54; 95% CI 0.09–0.81).

**Table 4:** Changes in Echo derived markers of right ventricular function.

	Sea Level	GHA	NH	HH	P Value
Right ventricular systolic pressure, mmHg					
-Rest	21.8 ± 4.0	31.8 ± 3.7	31.5 ± 5.8	31.7 ± 4.0	0.0002^abc^
-15 minutes post exercise	27.6 ± 5.2*	31.9 ± 6.1	31.4 ± 5.7	35.2 ± 5.7	0.04^c^
-2 hours post exercise	28.2 ± 4.0**	32.0 ± 6.4	32.0 ± 5.6	36.1 ± 8.1	0.0006^cf^
Pulmonary vascular resistance dynes/cm^5^					
-Rest	86.5 ± 13.8	107.5 ± 12.2	106.1 ± 15.9	118.7 ± 15.1	0.0002^a,b,c^
-15 minutes post exercise	111.5 ± 11.3*	116.8 ± 20.1	118.8 ± 19.3*	127.3 ± 13.8	0.11
-2 hours post exercise	106.7 ± 12.8**	114.5 ± 22.3	117.2 ± 15.6	129.1 ± 12.2	0.04^c^
TAPSE, cm					
-Rest	2.6 ± 0.4	2.2 ± 0.3	2.4 ± 0.3	2.6 ± 0.3	0.05
-15 minutes post exercise	2.3 ± 0.4*	2.3 ± 0.3	2.4 ± 0.3	2.5 ± 0.3	0.63
-2 hours post exercise	2.3 ± 0.3**	2.5 ± 0.3**	2.4 ± 0.3	2.6 ± 0.3	0.27
Tricuspid E velocity cm/s					
-Rest	56.2 ± 8.3	63.4 ± 18.1	62.2 ±12.9	66.2 ± 11.5	0.43
-15 minutes post exercise	50.4 ± 9.1	58.2 ± 12.5	50.8 ± 9.6*	57.7 ± 9.0	0.16
-2 hours post exercise	53.9 ± 10.2	58.5 ± 14.0	58.4 ± 10.5	54.3 ± 10.6 **	0.67
Tricuspid A velocity cm/s					
-Rest	33.7 ± 5.2	37.6 ± 1.0	45.9 ± 9.6	43.3 ± 10.0	0.01^b^
-15 minutes post exercise	39.9 ± 9.0*	45.8 ± 12.3*	51.5 ± 13.1	45.2 ± 13.7	0.16
-2 hours post exercise	35.4 ± 8.4	40.4 ± 7.4	51.7 ± 16.0	40.2 ± 7.1	0.006^b^
Right ventricular S’ velocity, cm/s					
-Rest	14.7 ± 1.3	15.5 ± 1.1	15.4 ± 1.6	14.5 ± 2.8	0.45
-15 minutes post exercise	13.2 ± 1.7	15.4 ± 1.5	13.4 ± 1.8*	15.2 ± 1.9	0.009^a^
-2 hours post exercise	13.9 ± 1.9	16.4 ±2.0	14.3 ± 2.3	14.8 ± 2.9	0.03^a^
Right ventricular E’ velocity, cm/s					
-Rest	14.2 ± 2.9	16.4 ± 3.5	14.8 ± 2.8	15.7 ± 2.9	0.30
-15 minutes post exercise	13.3 ± 3.8	16.6 ± 3.8	15.3 ± 4.7	15.3 ± 4.5	0.23
-2 hours post exercise	13.7 ± 3.0	16.4 ± 2.7	15.4 ± 3.3	15.2 ± 5.5	0.23
Right ventricular A’ velocity, cm/s					
-Rest	11.1 ± 1.9	11.5 ± 2.1	10.2 ± 1.9	12.2 ± 1.5	0.16
-15 minutes post exercise	10.9 ± 2.5	17.5 ± 3.1*	13.2 ± 4.1*	14.8 ± 1.5	0.0001^ad^
-2 hours post exercise	12.5 ± 2.4	18.1 ± 3.7**	14.9 ± 3.3*	14.8 ±1.3**	0.0007^a^
Right ventricular ICV velocity, cm/s					
-Rest	9.5 ± 2.7	10.5 ± 2.3	8.9 ± 1.9	9.1 ± 1.6	0.43
-15 minutes post exercise	10.8 ± 2.7	12.5 ± 3.1	10.2 ± 2.7	11.7 ± 2.1*	0.14
-2 hours post exercise	11.3 ± 3.1	12.2 ± 3.3	10.6 ± 1.9	9.7 ± 1.8	0.24
Right ventricular Tei Index					
-Rest	0.29 ± 0.05	0.32 ± 0.04	0.29 ± 0.03	0.30 ± 0.06	0.29
-15 minutes post exercise	0.33 ± 0.04	0.37 ± 0.05*	0.33± 0.03	0.41 ± 0.05*	0.001^cf^
-2 hours post exercise	0.32 ± 0.06	0.31 ± 0.05	0.34 ± 0.07**	0.39 ± 0.05**	0.04^e^

NN, normobaric normoxia; GHA, genuine high altitude; NH, normobaric hypoxia; HH, hypobaric hypoxia; TAPSE, trans annular plane systolic excursion; ICV, isovolumic contractile velocity; Between group post-test differences: a, NN vs GHA; b, NN vs NH; c, NN vs HH; d, GHA vs NH; e, GHA vs HH; f, NH vs HH. Within groups repeated measures post-test difference

* rest versus 15 minutes post exercise

** rest versus 2 hours post exercise.

### Two-Way Repeated Measures ANOVA comparing the three hypoxia groups

The main effects of hypoxia and time and the potential interactions (mode of hypoxia x time) and post tests are shown in Table 5 for the three hypoxia groups with NN as a reference. There was a significant main-effect for mode of hypoxia on SpO_2_, Tricuspid A, A’ and ICV as well as the RV Tei index (Table 5).

**Table 5:** Results of Two-Way Repeated Measures ANOVA comparing the Main Effects of Duration (time) and mode of hypoxia across the three hypoxia groups.

	Mode of hypoxia		Time		Interaction	
	F	P value	F	P value	F	P Value
SpO_2_	6.55	0.004^ac^	2.4	0.10	1.2	0.34
Heart Rate	2.32	0.12	75.47	<0.001^abc^	2.18	0.08
Mitral E velocity	0.17	0.84	11.97	<0.001^ac^	1.2	0.32
Mitral A velocity	1.20	0.33	5.9	0.005^ac^	0.35	0.85
Mitral E/A ratio	1.93	0.16	10.79	<0.0001	0.43	0.79
Septal S’ velocity	2.38	0.11	0.99	0.38	0.72	0.58
Septal E’ velocity	0.20	0.82	3.37	0.04^b^	0.77	0.55
Septal A’ velocity	0.29	0.75	12.32	<0.001^ab^	0.36	0.83
Septal ICV	1.0	0.40	2.70	0.08	1.0	0.42
Lateral S’ velocity	1.40	0.27	10.28	<0.001^ab^	3.99	0.006
Lateral E’ velocity	2.20	0.13	7.56	0.001 ^ab^	1.12	0.36
Lateral A’ velocity	0.40	0.68	6.97	0.002^ab^	0.58	0.68
Lateral ICV	0.12	0.89	6.99	0.002^a^	2.14	0.09
LV Tei Index	2.32	0.12	6.48	0.003^a^	1.54	0.10
LV stroke volume	0.39	0.68	10.37	0.004 ^ab^	0.93	0.45
Cardiac output	0.23	0.80	13.8	<0.001^ab^	2.52	0.06
RVSP	0.70	0.51	1.47	0.24	0.88	0.48
Pulmonary vascular resistance	2.10	0.14	5.85	0.005 ^ab^	0.27	0.76
Tricuspid E velocity	0.55	0.58	5.15	0.009^a^	0.88	0.48
Tricuspid A velocity	3.90	0.03^a^	1.9	0.16	0.58	0.68
TAPSE	1.21	0.31	1.1	0.31	2.4	0.06
Tricuspid S’ velocity	2.60	0.09	1.3	0.30	3.12	0.02
Tricuspid E’ velocity	0.66	0.53	0.01	0.99	0.10	0.98
Tricuspid A’ velocity	8.20	0.002^a^	22.3	<0.001 ^ab^	1.59	0.19
Tricuspid ICV	3.40	0.048 ^a^	6.7	0.002^a^	0.78	0.54
Right ventricular Tei Index	5.0	0.01^c^	16.10	<0.001^ab^	4.10	0.006

TAPSE, trans annular plane systolic excursion; RVSP, right ventricular systolic pressure; ICV isovolumic contractile velocity; Results of post hoc tests–time: a baseline versus 15 minutes post exercise, b baseline versus 2h post exercise, c 15 minutes versus 2 hours post exercise, altitude: a GHA vs NH, b GHA vs HH, c NH vs HH

There was a significant main effect for time on heart rate, mitral E and A, septal E’ and A’, lateral S’, E’, A’ and ICV, left ventricular Tei index and stroke volume, tricuspid E, A’ and ICV and RV Tei Index. Within group comparisons of the three hypoxic groups revealed no significant interactions between exercise and time for the majority of echo parameters. However there was a significant mode of hypoxia x time interaction effect for the lateral S’ (F [2, 56] = 3.99; p = 0.006: *n*^*2*^ = 0.22), tricuspid S’ (F [2, 58] = 3.12; p = 0.02: *n*^*2*^ = 0.16) and the right ventricular Tei index (F [2,58] = 4.1; p = 0.006; *n*^*2*^ = 0.23) (Figs 1–3, Table 5). The marginal means were consistently higher at 15 minutes post exercise with GHA and HH than NH (Figs 1–3).

## Discussion

This is the first study to assess the comparative changes in physiological and cardiac responses to exercise under four differing altitude conditions of NN, GHA, NH and HH. The key findings were that whilst all three hypoxic environments (GHA, NH and HH) led to similar cardiac adaptations at rest notable differences emerged following exercise. Compared with NH, the RVSP and RV Tei indices were higher with HH and the tricuspid A’ was higher with GHA. The degree of hypoxemia was greater with GHA and HH than with NH at both rest and at 15 minutes post exercise. There were no significant interactions between experimental altitude and time with the exception of the RV performance (Tei index) and the RV (tricuspid) and lateral S’ velocities.

There has been considerable debate in the literature as to whether differing modalities of hypoxia challenge are synonymous [2–7,25]. This is an important issue that remains unresolved and has enormous implications for HA research where inferences are often made from sea level chamber or hypoxia studies about potential responses at genuine HA. There have been only two studies, to date, that have assessed the effects of differing hypoxic environments on cardiac function. Miyagawa et al investigated seven young men who cycled for 40 minutes at 50% peak aerobic power in NN, NH and HH equivalent to 3200m in an artificial climate chamber [18]. Hence, this was a smaller sample size but similar altitude to our current study. Cardiac output and stroke volume were the only specific cardiac functional assessments performed beyond heart rate and were undertaken using pulse dye densitometry using Indocyanine Green [18]. However, both the reliability and reproducibility of this method has been challenged and its comparison to

more invasive methods of cardiac output determination have yielded conflicting results [26,27]. In their study, Miyagawa et al did not find any significant effects of experimental condition (trial) on cardiac output or stroke volume but did note there was a significant interactive effect of [trial × time] both cardiac output and stroke volume, during exercise, (suggesting that their responses to exercise were significantly different between the experimental conditions [18]. This related to the finding of a marked increase in cardiac output and stroke volume in both hypoxic groups versus the NN rather than any observed differences between the two hypoxic groups. Hence, as well as looking at a four-way comparison of the NN, GHA, NH and HH we undertook an additional exploratory analyses of the effects of hypoxia duration (time) and mode and their potential interactions across the three hypoxia groups. In another previous study two separate groups of six subjects were compared following approximately two hours of resting exposure to NH and HH at the equivalent of 4400m [17]. The only cardiac functional assessment performed was cardiac output, which was measured non-invasively using finger pulse waveform analysis [17]. No between group differences in cardiac output were observed however cycling time trial performance was worse with HH than NH.

In our study we assessed both right and left ventricular systolic and diastolic performance as well as markers of pulmonary artery haemodynamics (RVSP and the PVR) and global biventricular function using the Tei Index which adds significant novelty. Moreover, we ensured that the same exercise work and duration was maintained between the groups to reduce the confounding factor of differing exercise burden on any observed results.

One of the most pertinent findings of our study is the observation that it is not only the hypoxic environment but also exercise in this environment, which influences the cardiac and pulmonary vascular responses. Hence, ‘resting’ comparisons do not adequately reflect the reality of exposure to HA where there is usually an exercise component. This fact is supported in this study by the interaction between experimental conditions and exercise time for the Tei index and lateral left ventricular and right ventricular (tricuspid) S’ velocities (Figs 1–3). Whilst no significant between hypoxic group differences were observed over the short duration at rest, several differences emerged following exercise. For example, the RV Tei index and S’ velocities and the lateral left ventricular S’ velocities were consistently higher at 15 minutes post exercise with GHA and HH than NH. The higher values of RVSP, right ventricular Tei index and PVR and lower SpO_2_ post exercise with HH compared with NH are particularly notable in this study. In a very recent systemic review of crossover trials of HH versus NH, Coppel et al (2015) noted that peripheral SpO_2_ levels were higher with NH in two out of three short studies involving a hypoxic duration of <30 minutes with no notable differences in studies of ≥ 8 hours [2]. The authors also noted that arterial blood saturations (SaO_2_) were lower with HH in all three of the previously published short-term hypoxia-duration studies [2]. Our data is consistent with this limited published literature as we observed significantly lower SpO_2_ with HH than with NH. The suggested potential mechanisms to explain these observed differences, include lower minute ventilation, greater intravascular bubble formation and ventilation/perfusion mismatch, increased alveolar dead space as well as differences in alveolar fluid permeability and chemosensitivity with HH versus NH [2, 28].

Another factor, which must be considered is the effect of ambient temperature on SpO_2_ readings with finger pulse oximetry. It has been shown that a significant reduction in ambient temperature leads to peripheral vasoconstriction and can lead to a small (≤1.4%) increase in SpO_2_ which is thought to be explained by temperature-dependent arteriovenous shunts in the periphery [29,30]. Variations in core temperature can also affect the SaO_2_ (and hence SpO_2_) by rightward shift of the HbO2 dissociation curve [31]. In our current study, we tried maintain a similar exercise intensity and ambient temperature across all four experimental conditions. However, the ambient temperature was marginally, albeit significantly higher with HH (+2–3°C) versus NH and GHA and unfortunately, we did not record core temperature. Nevertheless, previously published studies have not identified any significant differences in core temperature and thermoregulation between NH and HH [2]. In either case, we would not expect this small variation in ambient temperature across the differing experimental conditions to lead to meaningful differences in core temperature and SaO_2._ Nevertheless the small temperature differences between experimental conditions is still a limitation that should be acknowledged. The fact that SpO_2_ was lower and PVR and RVSP were higher with GHA and HH than for NH strengthens our findings, given the well-established reciprocal relationship between SpO_2_ and RVSP/PVR, due to hypoxia driven pulmonary arterial vasoconstriction [10,20,32].

The observation of a greater increase in the RV Tei index with HH versus NH is an interesting and novel finding. The myocardial (Tei) index is a marker of global myocardial performance, which includes both systolic and diastolic functional parameters in its assessment and is independent of heart rate and ventricular geometry [23]. It may be a more sensitive marker of myocardial function than many traditional indices of cardiac function with increasing and higher values (>0.40–0.45) indicating worsening cardiac performance [23, 33]. The RV Tei index has been shown to positively correlate with both mean pulmonary artery pressure and PVR in patients with pulmonary artery hypertension and is highly susceptible to the effects of treatment [33,34]. The baseline (0.29–0.32) and post exercise increase in the RV Tei Index (up to 0.41) in our study is consistent with the published literature [33–35]. Huez et al previously noted that the RV Tei Index increased by approximately 50% (versus up to 33% in our study) following acute but more prolonged exposure to a higher altitude of 3750 among 15 healthy Caucasian adults [36]. More recently Page et al noted that the RV Tei Index increased significantly at genuine field HA (0.32±0.08 at 30 m to 0.43±0.15 at 3450m and 0.41±0.10 at 4730 m; P = 0.046) and was associated with subclinical pulmonary in 13 out of the 14 subjects [37]. This increase in the Tei Index was nearly identical to that in our study that is also in keeping with that noted among patients with treatment responsive pulmonary hypertension (22,33). We noted an interaction between exercise and HA environment on the RV Tei. (Fig 3). It has been previously demonstrated that heavy exercise under hypoxia leads to increased capillary disruption and fluid leak, which may be one of the mechanisms in the development of high altitude pulmonary oedema [38]. We observed that the PVR positively correlated with the RV Tei index and interestingly the HH group had both the highest Tei index and increase in PVR and PASP strengthening our findings. Our data suggests that increased PVR may be a have a negative effect on RV function, perhaps by increasing RV afterload due to increased PASP. The RV Tei Index appears to be particularly sensitive to even short-term changes in the hypoxic environment supporting previous work [33, 34].

The interaction between time and experimental conditions on the left and right ventricular S’ velocity appeared to relate to a more sustained increase in long axis function with GHA in relation to NN and NH where the changes were less marked (Figs 1 and 3). We also observed marked differences in the right ventricular systolic (S’ and ICV), diastolic (E and A’) and global function (Tei) depending on the hypoxic environment.

This study has a number of limitations that need acknowledgment. The sample size in the HH was smaller than among the other three groups, which could lead to selection bias and reduced power to detect a difference that was not appreciated. However, the demographics on the smaller HH group were similar to the other three groups and this sample size is at least as large as several previous comparative studies. Despite allowing for a reasonable ‘washout’ period between studies, it is uncertain whether the order of the studies could have influenced symptom scores and cardiac performance over time due to changes in physical fitness and experience of HA exposure. Furthermore, the effects of hyobaria was not independently assessed in this study through a hypobaric normoxia condition, as this was not feasible, and as such, the findings of this study should be reviewed in the context in which they have been presented.

In conclusion, HH, NH and HH produce similar cardiac adaptations at rest. However, notable differences emerge following exercise in the degree of hypoxemia, RVSP, RV systolic, diastolic and global function. This was most marked with HH and GH versus NH where the post exercise RV Tei and S’ velocity respectively were greater. The type of hypoxic environment and exercise performed in this environment significantly influence the cardiac response. Observed changes in cardiac function with NH are not necessarily predictive of similar changes with genuine HA or HH and vice versa.

## Supporting Information

S1 Table SPSS file with information on the three hypoxia groups and their respective echo data. (SAV)

S2 Table Excel file with full data for all four experimental study groups. (XLSX)
